# Surface‐Stabilized and Lightweight Metallic PET Fabrics for Flexible and Energy‐Dense Li‐Ion Batteries

**DOI:** 10.1002/advs.202513494

**Published:** 2025-09-23

**Authors:** Wancheng Yu, Zhenyao Wei, Lei Wang, Jian Shang, Hailong Xu, Yufeng Luo, Jiehua Cai, Chuan Xie, Yanpeng Guo, Junhua Zhou, Yonghong Deng, Qiyao Huang, Zijian Zheng

**Affiliations:** ^1^ Department of Applied Biology and Chemical Technology The Hong Kong Polytechnic University Hong Kong SAR 999077 China; ^2^ Laboratory for Advanced Interfacial Materials and Devices School of Fashion and Textiles The Hong Kong Polytechnic University Hong Kong SAR 999077 China; ^3^ Department of Materials Science and Engineering Guangdong Provincial Key Laboratory of Energy Materials for Electric Power Southern University of Science and Technology Shenzhen 518055 China; ^4^ Research Institute for Intelligent Wearable Systems The Hong Kong Polytechnic University Hong Kong SAR 999077 China; ^5^ Research Institute for Smart Energy The Hong Kong Polytechnic University Hong Kong SAR 999077 China

**Keywords:** current collector, flexible battery, lithium‐ion battery, metallic textile, surface stabilization

## Abstract

Current collectors are indispensable components in flexible lithium batteries. However, commercially used current collectors are heavy and rigid, severely limiting the energy density and flexibility of the batteries. Metallic polyethylene terephthalate fabrics (MPETs) have emerged as promising alternatives due to their lightweight nature, low cost, and excellent flexibility. Despite these advantages, the chemical and electrochemical stability of MPETs under battery operating conditions remains largely unexplored. Herein, the rapid degradation mechanism of MPETs in working batteries and propose effective surface‐stabilization strategies to enhance their long‐term stability is systematically investigated. An electroplating‐repair method is developed to fabricate etching‐proof MPETs for anodes, and a phosphorus‐incorporated nickel coating on PET to achieve high‐voltage‐stable MPETs for cathodes. Compared to commercial metal‐foil current collectors, the surface‐stabilized MPETs are significantly lighter –by 72.0% for the cathode current collector and 35.7% for the anode current collector, resulting in a 20% increase in battery energy density. FLBs assembled with these advanced MPETs exhibit outstanding cycling stability and maintain consistent voltage output even after thousands of bending cycles at radii as small as 1 mm. These results highlight the potential of surface‐stabilized MPETs to enable the next generation of energy‐dense and mechanically robust flexible lithium batteries.

## Graphical abstract

The electrochemical stabilities of metallic polyethylene terephthalate (MPET) current collectors. The pouch cell incorporating MPETs demonstrates a 20% increase in energy density, along with remarkable flexibility. It maintains a capacity retention of 78.4% after 1500 bending cycles under curvature radii as small as 1 mm.

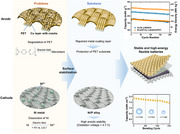

## 1 Introduction

The electronic industry is experiencing an evolution that changes rigid systems to flexible ones. Advancements in flexible electronics, such as roll‐up displays, soft robots, smart textiles, and implantable monitors, have spurred an increasing demand for flexible energy storage devices.^[^ ^1^, ^2^ ^]^ Among different technologies, flexible lithium batteries (FLBs) show significant potential for being reliable power support.^[^ ^3^, ^4^, ^5^, ^6^, ^7^, ^8^ ^]^ The flexibility and energy density of this type of battery largely depend on the mechanical property and areal density of current collectors (CCs). To date, commercial CCs of lithium‐ion batteries are copper (Cu) foils for the anode and aluminum (Al) foils for the cathode. Being indispensable components of a battery, these metal foils are rigid and easy‐to‐fatigue, significantly impeding the flexibility of the battery.^[^ ^9^, ^10^ ^]^ Additionally, these heavy metal foils are inactive components in energy storage, yet they contribute significantly to the battery's weight. Therefore, both the industry and research communities have been making strong efforts to develop alternative CC materials that are lightweight and intrinsically flexible, while maintaining similar processing ability in battery manufacturing and electrochemical/chemical stability in a running battery.

Replacing the metal foils with various kinds of carbon materials, such as carbon nanotubes,^[^ ^11^, ^12^, ^13^ ^]^ graphene,^[^ ^14^, ^15^, ^16^ ^]^ carbon felt,^[^ ^17^, ^18^, ^19^, ^20^ ^]^ and polymer‐derived carbon^[^ ^21^, ^22^ ^]^ are reported to effectively improve the battery's flexibility. However, their widespread industrial application is hindered by high costs, complex fabrication processes, and relatively high sheet resistance. In recent years, metallic fabrics have been considered as more promising candidates due to their superior strength, conductivity, interfacial adhesion with electrode materials, and rate performance.^[^ ^23^ ^]^ Carbon cloths and cotton fabrics were widely used to fabricate flexible CCs by taking advantage of their hierarchical woven structures.^[^ ^24^, ^25^, ^26^, ^27^, ^28^, ^29^ ^]^ Aiming for enhanced energy density, thinner and lighter glass‐fiber fabrics^[^ ^30^ ^]^ were used. Similarly, metallic polyethylene terephthalate fabrics (MPETs) were also utilized in several works as CCs for FLBs.^[^ ^31^, ^32^ ^]^ Among all, PET fabrics show advantages in cost, mechanical property, and areal density (**Table** **1**). Moreover, further decreasing the weight and thickness of PET fabrics is relatively easy to realize in the future because of its mature fabrication technology.

**Table 1:** Comparison of overall performance between different fabric substrates.

Substrate	Price [$ m^−2^]	Thickness [µm]	Areal density [mg cm^−2^]	Tensile strength [MPa]	Elongation at break [%]	References
PET fabric	0.1–0.3	12	1.2	≈150	≈30%	This work
Glass‐fiber fabric	0.5–1	20	2.1	≈150	≈3%	[30]
Cotton fabric	0.3–0.9	500	69.8	/	/	[33]
Carbon cloth	50–200	210	12.5	≈8	≈10%	[34]

**Figure 1:**
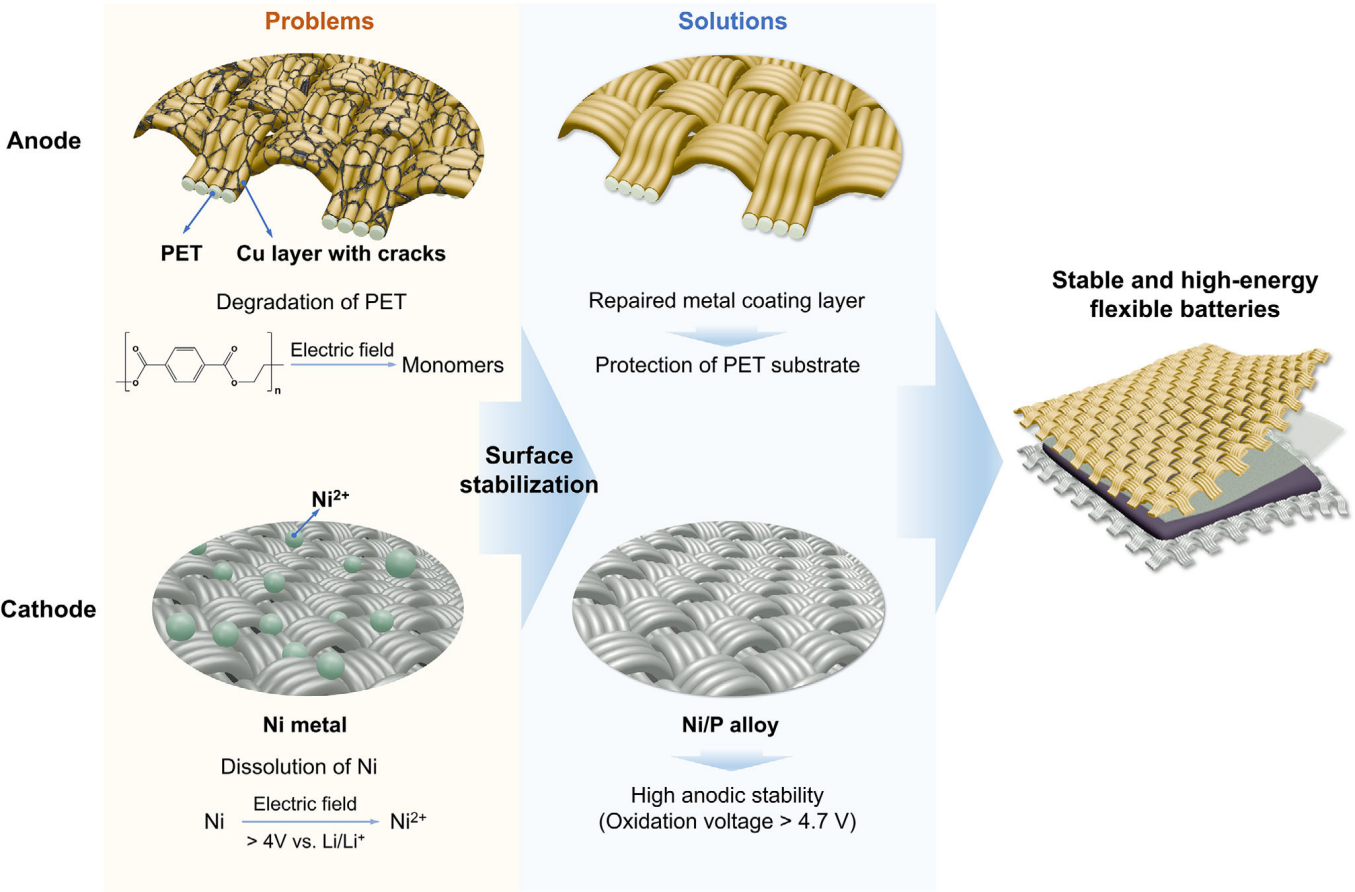
Problems and solutions of metallic PET (MPET) current collectors (CCs) in the anode and the cathode of the flexible lithium battery (FLB).

Typically, Cu‐coated PET fabrics (CuPETs) and Ni‐coated PET fabrics (NiPETs) were developed for the anode and the cathode of FLBs, respectively. While these MPETs excel in mechanical flexibility, ease of fabrication, and promising electrical conductance as CCs in FLBs, they exhibit shortcomings in chemical and electrochemical stability that have been underreported. On the anode side, PET may be involved in side reactions under low voltage in the discharge process because of the potential reduction of the ester group. As a result, the exposure of the PET fabric to the electrolyte resulting from the imperfect metal coating can significantly affect the battery performance (**Figure** **1**). On the cathode side, the Ni layer deposited on the NiPET will be oxidized to Ni^2+^ at potentials above 4.0 V versus Li/Li^+^ (Figure 1).^[^ ^35^, ^36^ ^]^ The resulting electrochemical window, however, cannot meet the demands for high‐energy cathode materials, such as lithium cobalt oxides (LCO) and Lithium nickel cobalt manganate (NCM), which typically necessitate a voltage window of at least 4.2 V. Therefore, it is crucial to stabilize the MPETs to enhance their compatibility and performance as CCs in high‐energy FLBs.

Herein, we report surface‐stabilized MPETs as CCs for energy‐dense FLBs. Building upon the electrochemical degradation mechanism of MPETs in the anode and cathode, we propose an electroplating‐repair method for CuPETs (ECuPET) and an alloying strategy for NiPETs (NiPPETs), respectively. For the former, further electroplating of Cu onto CuPET effectively repairs the imperfect Cu coating layer on the metallic fabric, offering a dense protective layer to the PET fabric to eliminate its exposure to the electrolyte. For the latter approach, the incorporation of phosphorus (P) during the metal deposition offers a Ni/P alloy coating layer for the PET fabric, promoting the anodic stability. These two approaches effectively surface‐stabilize the MPETs, enabling a significant enhancement in electrochemical stability and the expanded charge/discharge window beyond 4.7 V. As a result, the pouch cell using these surface‐stabilized MPETs as CCs exhibits outstanding cyclic stability with a capacity retention of 90.6% after 200 charge–discharge cycles. The lightweight properties of MPETs (2.1 mg cm^−2^ for ECuPET and 2.7 mg cm^−2^ and NiPPET) significantly surpass those of metal foils (7.5 mg cm^−2^ for Cu foil and 2.7 mg cm^−2^ for Al foil), resulting in a 20% improvement in energy density for the battery. While MPETs elevate the energy performance of the lithium battery to a level comparable to that of conventional counterparts based on metal foils, they endow the cell with exceptional mechanical flexibility. The MPETs‐based FLB showcases high capacity retention of 78.4% after undergoing 1500 bending cycles at the bending radii of 5, 2, and 1 mm. Moreover, it can work reliably through a charge‐discharge program synchronized with bending, even at a bending radius as low as 3 mm.

## 2 Results and Discussion

### 2.1 Enhancing Anode Stability with Electroplating‐Repaired CuPET (ECuPET)

**Figure 2:**
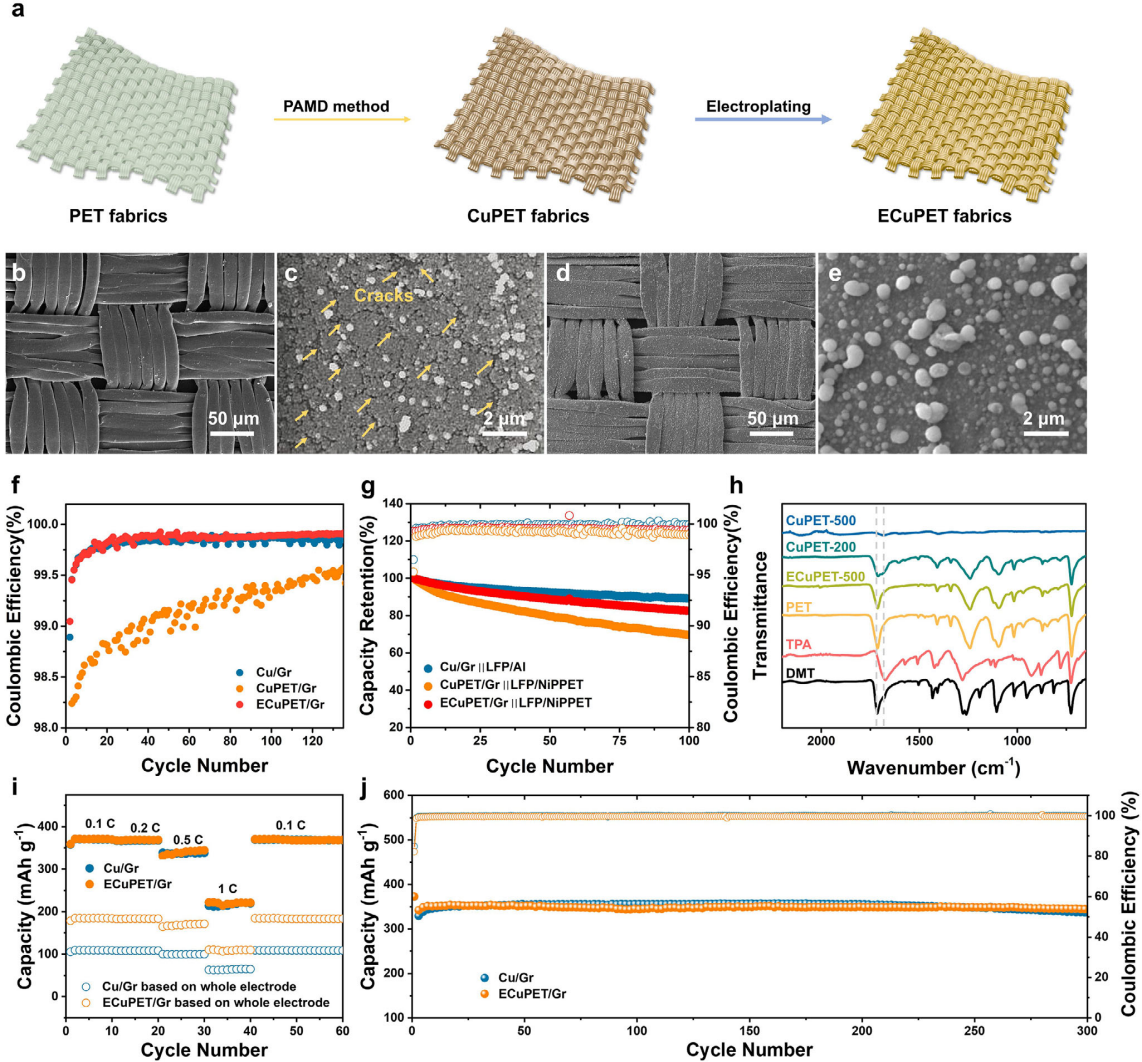
a) Schematic fabrication of CuPET and ECuPET. Scanning electron microscope (SEM) images of b,c) CuPET and c,d) ECuPET. f) Coulombic efficiencies (CEs) of Cu/Gr, CuPET/Gr, and ECuPET/Gr. g) Cyclic performance of cells of Cu/Gr||LFP/Al, CuPET/Gr||LFP/NiPPET, and ECuPET/Gr||LFP/NiPPET h) Fourier Transform Infrared Spectroscopy (FTIR) results of CuPET and ECuPET from the cycled batteries. i) Rate and j) cyclic performances of Cu/Gr and ECuPET/Gr. The mass loading of Gr is ≈5.4 mg cm^−2^.

CuPETs are commonly produced by metalizing PET with Cu.^[^ ^31^, ^32^, ^37^ ^]^ To ensure a robust adhesion between the Cu coating layer and the PET fabric, we initially adopted the polymer‐assisted metal deposition (PAMD) approach, a well‐developed solution‐processable metallization strategy, to achieve a conformal and even Cu coating (**Figure** **2**a).^[^ ^38^, ^39^ ^]^ Due to the granule‐stack deposition mechanism, which often leads to gaps between Cu granules during the electroless deposition (Figure S1, Supporting Information), CuPET fabricated by the PAMD approach usually experiences cracks on the plated Cu layer (Figure 2b,c). These cracks expose the PET fabric to the electrolyte, resulting in the chemical and electrochemical instability of the electrode over an extended operation period. Such an imperfect coating layer can be repaired by electroplating (Figure 2a). After the Cu electrodeposition, PET is enveloped in a fresh layer of Cu without any cracks (Figure 2d,e).

The electrochemical stability of CuPET and its electroplated counterpart (denoted as ECuPET) was assessed to underscore the importance of repairing the metal coating layer on CuPET CCs. They were both coated with graphite (Gr) to form the Gr anodes (denoted as CuPET/Gr and ECuPET/Gr) to investigate the electrochemical performance. The electrolyte of 1 m LiPF_6_ in ethylene carbonate (EC)/diethyl carbonate (DEC)/ethyl methyl carbonate (EMC) is used for all the electrochemical tests unless otherwise specified. As depicted in Figure 2f, while the Coulombic efficiency (CE) of CuPET/Gr is notably lower, ECuPET/Gr shows comparable CEs to the conventional Gr anode (i.e, graphite‐coated Cu foil (Cu/Gr)), implying the contribution of the electroplated Cu on the ECuPET CC toward the performance enhancement (Figure 2f). Such a performance enhancement effect intensifies with the electroplating time prolonged from 15 to 30 min (electroplating current density: 0.5 mA cm^−2^). However, further extension to 120 min shows a less noticeable impact on performance enhancement (Figure S2, Supporting Information). The final electroplating conditions (0.5 mA cm^−2^, 30 min) were selected to achieve an optimal balance between the protective efficacy and the efficient utilization of copper resources. The performance difference between CuPET and ECuPET is also evident in the full cell test: when paired with LFP, the cycling performance of CuPET/Gr is notably inferior to that of ECuPET/Gr (Figure 2g). These electrochemical tests showcase the role of the electroplated Cu layer in enhancing electrochemical stability during charge/discharge processes.

We attribute the performance enhancement in anodes with ECuPET CCs to the surface stabilization provided by the additional electroplated Cu layer, which effectively repairs the flaws in the pristine Cu coating that PAMD fabricated. As a result, the exposure of the PET substrate to the electrolyte is minimized, preventing undesired side reactions during the electrochemical charge/discharge cycles. Further validation through Fourier Transform Infrared Spectroscopy (FTIR) analysis of ECuPET and CuPET CCs after the cycling tests reveals such insights. As depicted in Figure 2h, the peak of the ─COO‐ functional group originally positions at 1710 cm^−1^ for CuPET shifts to lower wavenumbers after 200 charge/discharge cycles and eventually diminishes after 500 cycles. Such changes indicate that PET fibers within CuPET have undergone ester bond cleavage, leading to the generation of carboxylic derivatives (refer to the FTIR curves of TPA and DMT, two possible products). In contrast, there are no noticeable changes in the FTIR spectra of ECuPET after 500 charge/discharge cycles, demonstrating the outstanding stability of this surface‐treated fabric CC.

As a result, the surface‐stabilized ECuPETs stably perform their high electrical conductance as CCs during the charge/discharge processes by mitigating the negative impact of PET exposure to electrolytes on electrode stability. The resultant ECuPET/Gr has a rate capability similar to Cu/Gr, demonstrating a high capacity of ≈350 mAh g^−1^ at a rate of 0.5C (Figure 2h). It can retain up to 97.5% of its capacity after 300 charge/discharge cycles, which is comparable to that of Cu/Gr (Figure 2i). Notably, with a low areal density of 2.1 mg cm^−2^, ECuPET is significantly lighter than the Cu foil (7.5 mg cm^−2^). When considering the capacity based on the entire electrode (including the CCs), the capacity of ECuPET/Gr (185 mAh g^−1^ at 0.1 C) is nearly double that of Cu/Gr (109 mAh g^−1^ at 0.1 C). In addition, ECuPET is durable in long‐term bending test, showing no discernible cracks of copper layers after 10 000 bending cycles (Figure S3, Supporting Information).

To reveal the degradation mechanism of CuPET during the charge/discharge processes and validate the surface stabilization of additional electroplated Cu on CuPET, we first coat CuPET with a several‐nanometer‐thick Al_2_O_3_ layer by atomic layer deposition (ALD). It is hypothesized that the physical barrier offered by such Al_2_O_3_ layer can prevent the reactive chemicals in the electrolyte from interacting with PET (Figure S4a, Supporting Information), thereby enhancing the electrochemical stability of the CuPET/Gr electrodes. However, while the CEs of the ALD‐protected electrodes show a slight improvement, it still takes hundreds of charge/discharge cycles, a similar trend to CuPET/Gr, to reach a stable state for the electrodes (Figure S4b, Supporting Information). Furthermore, the specific capacities of ALD‐protected electrodes are lower in comparison to those of CuPET/Gr. (Figure S4c, Supporting Information). Such ineffective protection offered by Al_2_O_3_ physical battery suggests that Li^+^ may be involved in the side reaction because the protective layer is a Li^+^ conductor.

**Figure 3:**
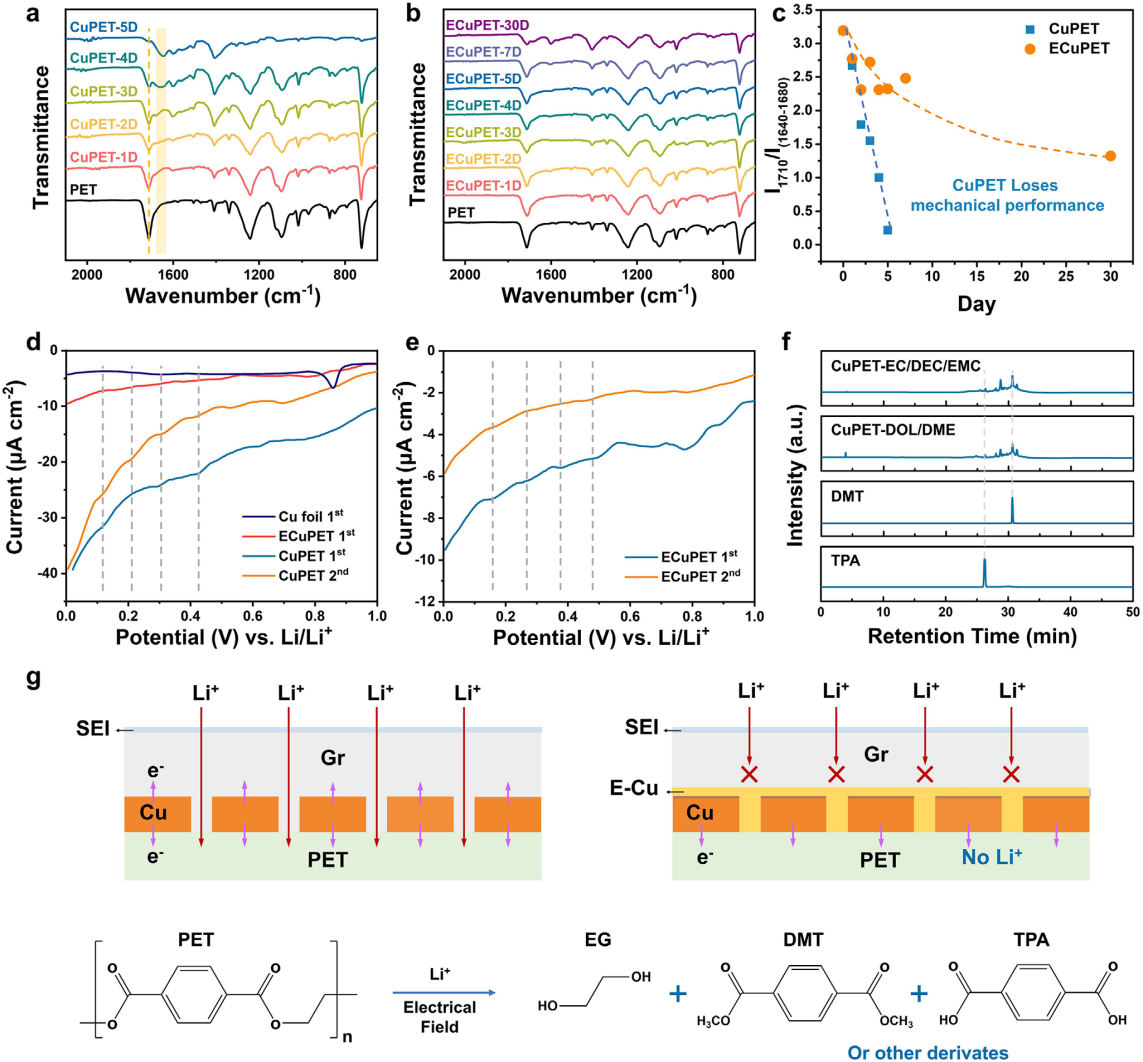
FTIR results of a) CuPET and b) ECuPET from the Li||CuPET (ECuPET) cells set at 0 V (vs Li/Li^+^) for different days. c) The intensity ratio of the peak at 1710 cm^−1^ to peaks at the range of 1640–1680 cm^−1^ as a function of time. d,e) CV curves of CuPET and ECuPET at a scan rate of 0.5 mV s^−1^. f) HPLC curves of CuPET set at 0 V (vs Li/Li^+^) in different electrolytes after seven days. g) Schematic degradation and protection mechanism of the PET substrate.

We then investigate the influence of the Cu coating layer on the PET degradation by placing pristine PET, CuPET, and ECuPET fabrics in direct contact with Li foils for several days and assessing their changes in material compositions (Figure S5a, Supporting Information). While pristine PET remains stable with Li foil, CuPET undergoes significant degradation after ten days (Figure S5b, Supporting Information). On the other hand, ECuPET demonstrates superior anticorrosion performance in comparison with CuPET. The degradation of PET intensifies during the electrochemical process (0 V vs Li/Li^+^) (Figure S6a, Supporting Information). After only one day, a shoulder peak at 1670 cm^−1^ appears for CuPET, with an increased intensity of the C─C peak on the benzene ring (**Figure** **3**a). After just five days, the ─COO‐ peak is almost vanished and the peaks of ester or carboxylic derivatives are located at the range of 1640–1680 cm^−1^. After six days, CuPET was almost pulverized and could not be tested anymore. In sharp contrast, ECuPET mainly maintains its original chemical structure in the first week (Figure 3b). After a long‐term test of a month, the degradation peak can be found for ECuPET. It should be noted that continuously staying at 0 V versus Li/Li^+^ is a harsh condition for the CC, as the accumulated time that a CC spends at 0 V is only a small fraction of the operating time in a commercial lithium battery. Given these conditions, ECuPET has shown reliable stability on the anode side, although the degradation of PET is not totally prevented. The extent of degradation of the PET substrate can be quantitatively determined by the change in the C═O peak, which is calculated as the intensity ratio of the peak at 1710 cm^−1^ to those in the range of 1640–1680 cm^−1^ (I_1710_/I_1640‐1680_) (Figure 3c). A linear decrease of I_1710_/I_1640‐1680_ is observed for CuPET, which quickly declines to 0.2 after only 5 days. On the contrary, the intensity ratio for ECuPET fluctuates from 2.3 to 2.7 in the first week.

Several stages on CV curves of CuPET and ECuPET suggest a multi‐stage degradation reaction of the PET (Figure 3d,e). It should be noted that the current density of ECuPET in the CV test is comparable to that of Cu foil and much lower than that of CuPET. The leakage current at 0 V versus Li/Li^+^ also proves that ECuPET has better electrochemical stability than CuPET (Figure S6b, Supporting Information). ECuPET shows a stable leakage current while CuPET presents higher current densities with fluctuations. The extra electron flow for CuPET is attributed to the side reaction between the PET and the electrolyte. Then, the degradation products are analyzed by high‐performance liquid chromatography (HPLC). Several esters or carboxylic derivatives, including the terephthalic acid (TPA) and dimethyl terephthalate (DMT) are generated (Figure 3f), corresponding well with the multi‐stage reaction in the CV test. As mentioned above, a chemical degradation process of PET may occur in the electrolyte containing DEC to produce DMT.^[^ ^40^, ^41^ ^]^ To exclude the influence of the alcoholysis process, an electrolyte of 1 m LiPF_6_ in 1,3‐dioxolane (DOL)/1,2‐dimethoxyethane (DME) electrolyte was applied. Similar products are generated in the DOL/DME electrolyte, which is confirmed by the HPLC, gas chromatography‐mass spectrometer (GC‐MS), and the FTIR results (Figure 3f; Figures S7 and S8, Supporting Information). These results manifest that electric‐field‐induced degradation is the dominant mechanism in this work. For CuPET, the PET substrate reacts with Li^+^ and generates several ester or carboxylic derivatives by the driving force of the electric field. After electroplating, the cracks and micro‐holes in the Cu layer are repaired, largely blocking the penetration of Li^+^. Although the Cu layer can still conduct electrons to PET, the side reactions are significantly hindered.

### 2.2 Enhancing Anodic Stability of Cathode with Phosphorous‐Incorporated NiPET (NiPPET)

**Figure 4:**
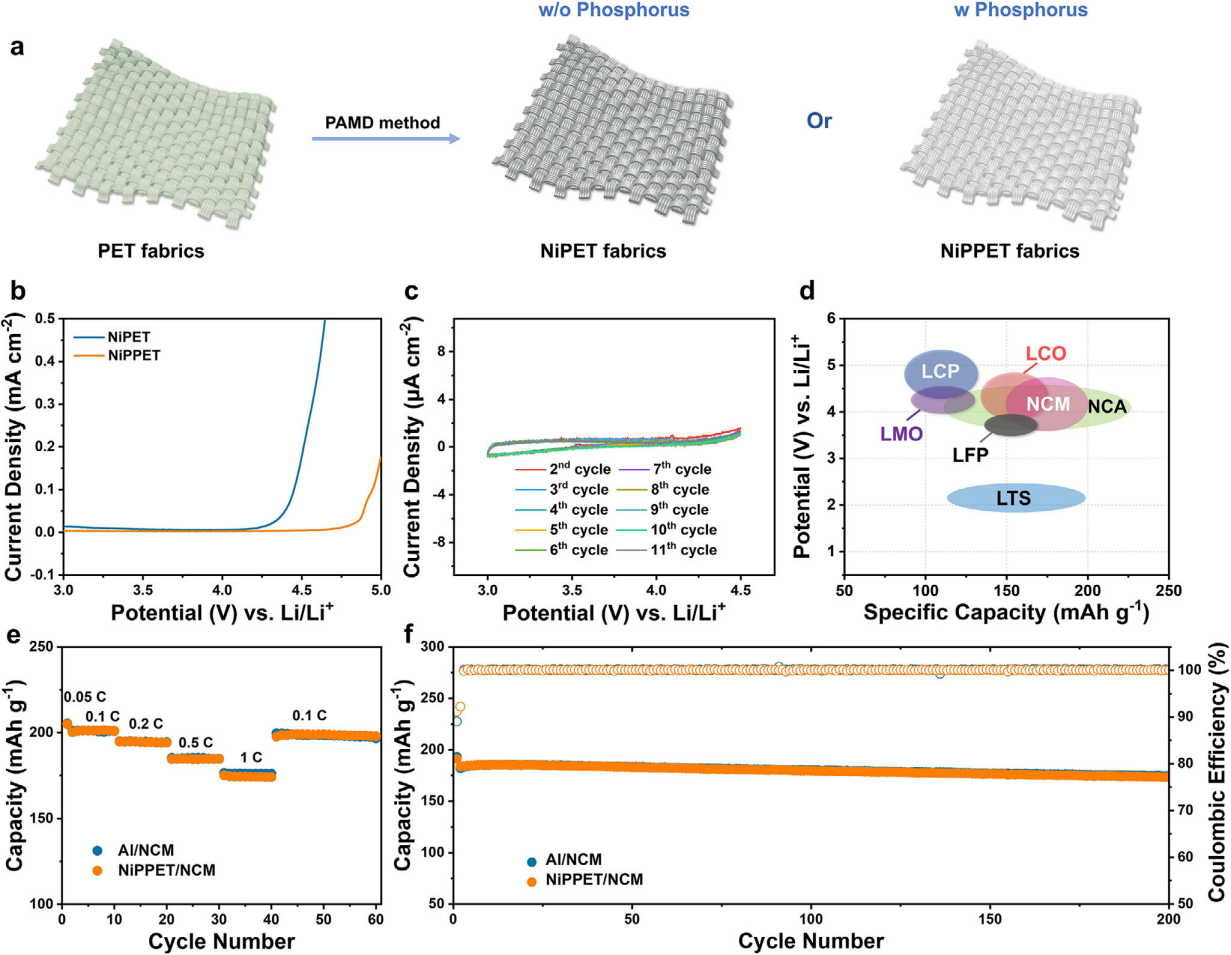
a) Schematic fabrication of NiPET and NiPPET. b) Linear scanning voltammetry (LSV) curves of NiPPET recorded at a scan rate of 0.5 mV s^−1^ in the range of 3–5 V (vs Li/Li^+^). c) CV curves of NiPPET recorded at a scan rate of 0.5 mV s^−1^ in the range of 3–4.5 V (vs Li/Li^+^). d) Cut‐off voltages and specific capacities of commercial cathode materials. e) Rate and f) cyclic performance of Al/NCM and NiPPET/NCM electrodes. The mass loading of NCM is ≈10.0 mg cm^−2^.

For the cathode side, Ni metal is widely utilized as a conductive coating layer for CCs. However, the anodic stability of pure Ni is not sufficient to meet the requirement of high‐voltage cathode materials commonly used in commercial Li batteries. Specifically, when tested in a cell, NiPET begins to oxidize at the potential of ≈4.0 V (vs Li/Li^+^), which is below the operating voltage of most commercial cathodes such as NCM (**Figure** **4**b).^[^ ^35^, ^36^ ^]^ Following only a single charge‐discharge cycle, the NiPET exhibits a pronounced color change to black, accompanied by a marked decrease in the intensity of Ni‐related peaks in the X‐ray photoelectron spectroscopy (XPS) spectra (Figure S9, Supporting Information). This early onset of oxidation limits the practical application of NiPET as a current collector for high‐voltage FLBs.

To overcome this limitation, phosphorus (P) herein is introduced during the PAMD process, resulting in the formation of a Ni/P alloy coating layer on the fibrous matrix of PET fabric (i.e., NiPPET) (Figure 4a; Figure S10, Supporting Information). Such a Ni/P alloy is simply formed by employing a Ni plating solution containing phosphorus, whereas the NiPET without Ni/P alloy coating is obtained using a P‐free plating solution. The incorporation of P effectively enhances the anodic stability of the metallic fabric CCs (NiPPET). Electrochemical measurements reveal that NiPPET exhibits a significantly higher onset anodic potential of ≈4.7 V (vs Li/Li^+^), which is much higher than that of NiPET (Figure 4b). Further cyclic voltammetry tests conducted in the range of 3–4.5 V (vs Li/Li^+^) demonstrate that NiPPET maintains excellent long‐term electrochemical stability within this potential window (Figure 4c). Such a stable electrochemical window is broad enough to accommodate the operating voltages of most cathode materials (Figure 4d), showcasing that NiPPET is well‐suited as CC for high‐voltage cathodes.

The practical electrochemical performance of NiPPET is evaluated by using NCM as the cathode materials. When compared to the conventional Al/NCM cathode, NiPPET/NCM exhibits nearly identical rate capability and cyclic performances. Specifically, NiPPET/NCM delivers a high capacity of 174 mAh g^−1^ at 1 C, corresponding to ≈86.5% of its capacity at 0.1 C (Figure 4e). Moreover, after 200 charge–discharge cycles, NiPPET/NCM retains 92.5% of its initial capacity, demonstrating excellent cycling stability (Figure 4f).

In summary, the introduction of P during the PAMD process leads to the formation of a Ni/P alloy coating on PET fabrics, which can significantly improve the anodic stability of the current collector. The enhanced electrochemical window and robust cycling performance of NiPPET make it a promising high‐voltage current collector for the cathode side, supporting the development of high‐performance Li batteries.

### 2.3 Synergistic Performance Enhancement of FLBs by MPETs

**Figure 5:**
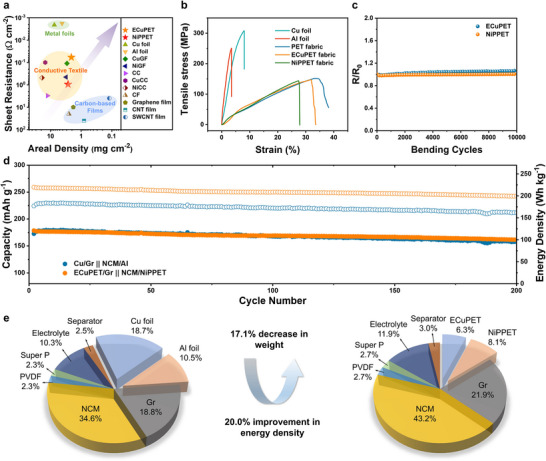
a) Comparison of areal density and sheet resistance among different CCs, including commercial metal foils and other reported flexible CCs. Detailed information is listed in Table S1 (Supporting Information). b) Typical stress‐strain curves for metal foils, original PET fabric, and MPETs. c) Resistance variations of MPETs during bending cycles at a bending radius of 2 mm. d) Cycle performance of the cells of Cu/Gr||NCM/Al and ECuPET/Gr||NCM/NiPPET. The mass loading of NCM and Gr are 11.1 and 7.3 mg cm^−2^, respectively. The computational details of energy densities are listed in Table S2 (Supporting Information). e) Comparison on weight fractions of conventional and MPET‐based batteries showing a distinct improvement in energy density. The computational details of energy densities are listed in Table S3 (Supporting Information).

MPETs, both ECuPET and NiPPET, exhibit outstanding synergistic properties compared to other reported CCs when applied in FLBs.[^11^, ^27^, ^30^, ^34^, ^42^, ^43^, ^44^] First, the uniform metal layers endow low sheet resistances to MPETs, with a value of ≈0.8 Ω cm^−2^ for NiPPET and ≈0.06 Ω cm^−2^ for ECuPET, respectively (**Figure** **5**a). Second, both ECuPET and NiPPET possess low areal densities −2.1 and 2.7 mg cm^−2^, respectively – which are significantly lower than those of metal foils (7.5 mg cm^−2^ for Cu foil and 4.2 mg cm^−2^ for Al foils). Moreover, MPETs simultaneously achieve high mechanical strength and excellent flexibility (Figure 5b). They demonstrate ultimate tensile strengths of ≈145 MPa, which is sufficient for roll‐to‐roll production. The elongations at break of these metallic fabrics are ≈30%, suggesting superior deformability compared to metal foils. The flexibility of the MPETs is further evaluated by cyclic bending tests, during which both ECuPET and NiPPET showed less than 5% variations in resistance (Figure 5c). The stable conductivity can be ascribed to the robust adhesion between the metal layer and substrate.

Motivated by the excellent electrical, electrochemical, and mechanical properties of MPET CCs, ECuPET/Gr and NiPPET/NCM are paired together to assemble a full battery with an N/P ratio of ≈1.1. The resulting cell demonstrates a capacity retention of 90.6% after 200 charge–discharge cycles, which is comparable to that of a battery utilizing conventional metal foils (Figure 5e). Owing to the lower areal densities of MPETs, the ECuPET/Gr||NCM/NiPPET cell achieves a high energy density of 220 Wh kg^−1^, representing a 20% increase over cells employing metal foil CCs (Figure 5e). Notably, this enhancement in energy density is simply achieved by replacing the CC without compromising electrochemical performance. Our MPETs exhibit a well‐balanced and remarkable overall performance compared to previously reported flexible CCs, demonstrating considerable potential for application in FLBs (Table S1, Supporting Information).

**Figure 6:**
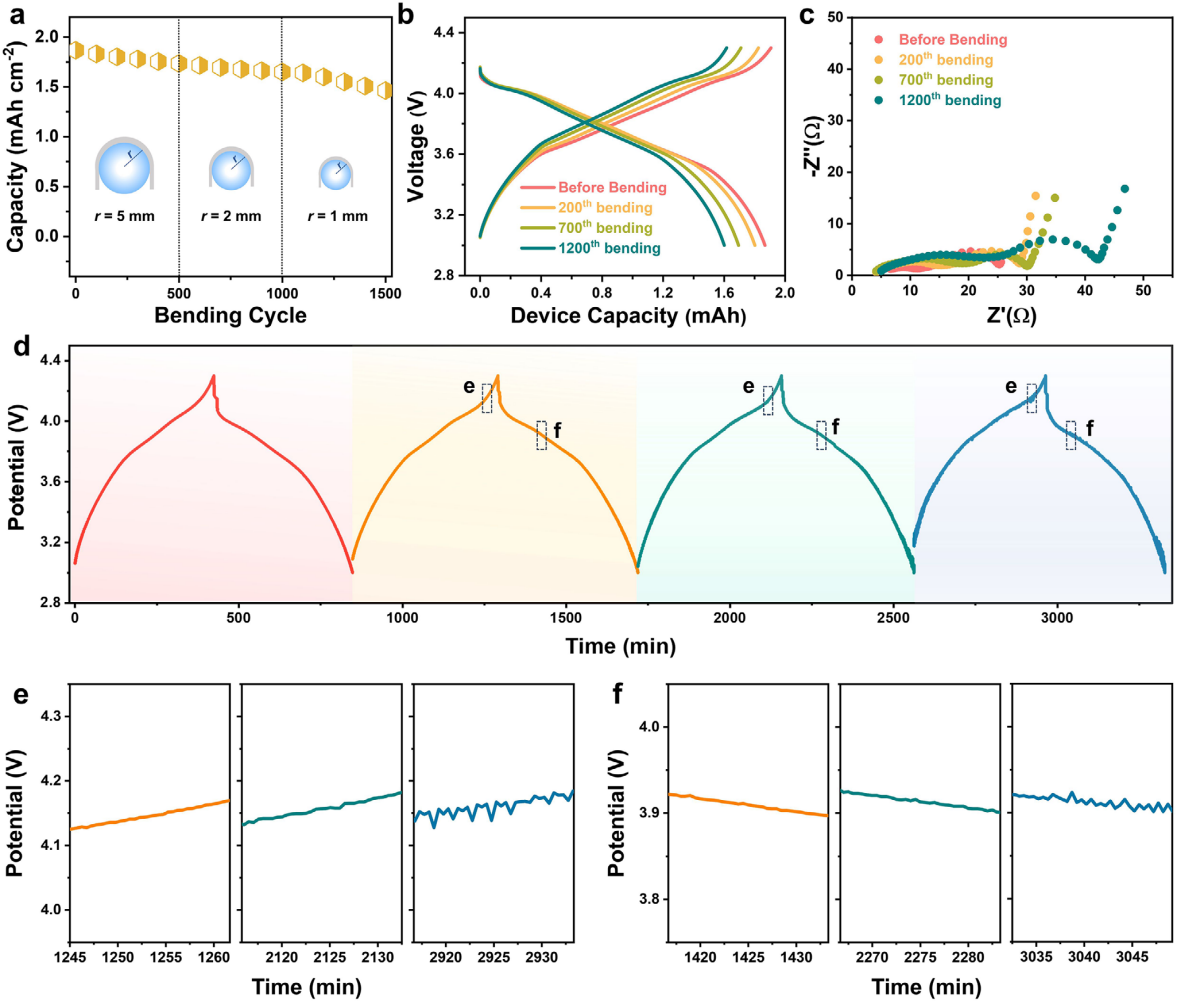
a) Areal capacity of the ECuPET/Gr||NCM/NiPPET pouch cell as a function of bending cycles at radii of 5, 2, and 1 mm. b) Corresponding GCD curves and c) EIS results of the pouch cell before and after bending cycles. d) GCD curves of ECuPET/Gr||NCM/NiPPET pouch cell at the flat state and during the continuous bending process at bending radii of 7, 5, and 3 mm. Enlarged curves in e) the charging process and f) the discharging process of the pouch cell. The mass loadings of NCM and Gr are ≈11.0 and 7.0 mg cm^−2^, respectively.

The flexibility of the MPET‐based pouch cell is evaluated through both ex situ and in situ tests (Figure S11, Supporting Information). In the ex situ flexibility test, the bending process is conducted separately from the charge/discharge cycles. Specifically, the pouch cell is first subjected to a charge/discharge cycling test, and then bent for 500 cycles at specified bending radii (5, 2, and 1 mm) and subsequently reconnected for further electrochemical measurements (**Figure** **6**a). The ECuPET/Gr||NCM/NiPPET pouch cell presents a high capacity retention of 88.4% after 1000 bending cycles, showing excellent mechanical robustness even under a harsh bending state with a bending radius of 2 mm. Under a smaller bending radius of 1 mm, the decay of capacity becomes slightly faster, resulting in a capacity retention of 78.4% after 1500 bending cycles. The corresponding galvanostatic charge/discharge (GCD) curves after breending manifest typical features of NCM materials with slowly increased overpotentials (Figure 6b).

EIS results of the ECuPET/Gr||NCM/NiPPET pouch cell at the flat state and after different bending cycles are recorded. Two semicircles that correlate to the R_inter_ (interface resistance) and R_ct_ (the resistance derived from the charge transfer) appear for all the samples. After bending at 5 and 2 mm, the R_inter_ increases slightly with a stable R_ct_ compared to the flat state (Figure 6c). The slight increase in interface resistance may be triggered by the partial detachment of active materials on the current collector. Even when bending at 1 mm for 500 cycles, rises on R_inter_ and R_ct_ are still acceptable, which explains the high capacity retention of the pouch cell during bending cycles.

For the in situ test, the pouch cell is continuously bent at the specified radius while simultaneously undergoing charge/discharge cycling. The bending radii are chosen as 7, 5, and 3 mm. The ECuPET/Gr||NCM/NiPPET pouch cell can work stably during the bending processes, delivering 1.76, 1.66, and 1.50 mAh cm^−2^ at bending radii of 7, 5, and 3 mm, respectively, which are comparable to the capacity of 1.73 mAh cm^−2^ observed in the flat state (Figure 6d). The potential fluctuations for pouch cell bending at 5 and 3 mm are minimal, as shown in the enlarged GCD curves (Figure 6e,f). The undulation of the potential becomes more pronounced when the pouch is bending at 3 mm but is still acceptable for a working battery. In addition, it can be seen that the fluctuations in the charge curve are smaller than those in the discharge curve. These results demonstrate that our MPET‐based FLBs exhibit high output stability even under rigorous bending conditions.

## 3 Conclusion

In summary, we have demonstrated that surface‐stabilized MPETs are highly promising CCs for high‐energy FLBs. By addressing the electrochemical degradation mechanisms of MPETs, we have developed an electroplating‐repair structure for CuPET and an alloying approach for NiPET, resulting in ECuPET and NiPPET with significantly enhanced surface stability. These modifications not only provide robust protection for the PET substrate and expand the electrochemical stability window beyond 4.7 V, but also enable the assembled pouch cells to achieve outstanding cycling performance, with a capacity retention of 90.6% after 200 charge–discharge cycles. The lightweight nature of MPETs contributes to a 20% increase in energy density compared to conventional metal foil‐based batteries. Importantly, MPET‐based FLBs inherit the excellent mechanical flexibility of PET fabrics, maintaining a high capacity retention of 78.4% even after 1500 bending cycles at radii as small as 1 mm. Moreover, these cells operate reliably under synchronized charge‐discharge and bending conditions, delivering stable performance even at a bending radius of 3 mm. Overall, these results highlight the potential of surface‐stabilized MPETs to enable the next generation of FLBs with both high energy density and exceptional mechanical durability, paving the way for their application in flexible and wearable electronic devices.

## Conflict of Interest

The authors declare no conflict of interest.

## Supporting information



Supporting Information

## Data Availability

The data that support the findings of this study are available in the supplementary material of this article.
