# Supplementary material for: Surface‐Stabilized and Lightweight Metallic PET Fabrics for Flexible and Energy‐Dense Li‐Ion Batteries
Source: Adv Sci (Weinh). 2025 Sep 23;12(46):e13494. doi: 10.1002/advs.202513494 (PMC12697824; doi:10.1002/advs.202513494)
Supplement: Supplementary file 1 — Supporting Information [file ADVS-12-e13494-s001.docx]

Supporting Information

**Surface Stabilization of Metallic PET Fabrics for Flexible and Energy-dense Li-ion Batteries**

Wancheng Yu, Zhenyao Wei, Lei Wang, Jian Shang, Hailong Xu, Yufeng Luo, Jiehua Cai, Chuan Xie, Yanpeng Guo, Junhua Zhou, Yonghong Deng, Qiyao Huang, Zijian Zheng^*^

Dr. W. Yu, Dr. Y. Luo, Dr. C. Xie, Dr. Y. Guo, Dr. J. Zhou, Prof. Z.J. Zheng

Department of Applied Biology and Chemical Technology, The Hong Kong Polytechnic University, Hong Kong SAR, China

E-mail: [tczzheng@polyu.edu.hk](mailto:tczzheng@polyu.edu.hk)

Dr. W. Yu, Dr. Z. Wei, Dr. L. Wang, Dr. J. Shang, Dr. H. Xu, Dr. J. Cai, Prof. Q. Huang, Prof. Z. J. Zheng

Laboratory for Advanced Interfacial Materials and Devices, School of Fashion and Textiles, The Hong Kong Polytechnic University, Hong Kong SAR, China

Prof. Z. J. Zheng

Research Institute for Smart Energy, The Hong Kong Polytechnic University, Hong Kong, SAR, China

Prof. Q. Huang, Prof. Z. J. Zheng

Research Institute for Intelligent Wearable Systems, The Hong Kong Polytechnic University, Hong Kong SAR, China

Dr. Z. Wei, Prof. Y. Deng,

Department of Materials Science and Engineering

Guangdong Provincial Key Laboratory of Energy Materials for Electric Power Southern University of Science and Technology

Shenzhen, 518055, China.

Keywords: current collector, metallic textile, flexible battery, surface stabilization, lithium-ion battery.

**Experimental Section**

*Materials and Chemicals.* All chemicals were purchased and used without further purifications unless specified. PET fabrics were received by Nantong Yanbo Chemical Fiber Co., Ltd., China. Ethyl Alcohol (EtOH, 99.9%) was supplied by AQA Co., Ltd., USA. Acetic acid (100%) and Lactic acid (90%) were provided by Acros Co., Ltd., USA. 3-(trimethoxylsilyl)propyl methacrylate (MPTS, 98%), [2-methacryloyloxy)ethyl]trimethylammonium chloride solution (METAC, 80ωwt %) and potassium sodium tartrate tetrahydrate (99.5%) were purchased from Sigma-Aldrich Co., Ltd., USA. Potassium persulfate (KPS, 99.5%), Nickel (II) Sulfate (AR), sodium hydroxide (97%) and copper(II) sulfate pentahydrate (99.0%) were offered by Uni-Chem Co., Ltd., India. Sodium hypophosphite (AR) and 1-methyl-2-pyrrolidinone (NMP) were purchased from Aladdin Co., Ltd., China. Formaldehyde solution (37% - 41%) and sodium acetate were provided by VWR International Co., Ltd., USA. Ammonium tetrachloropalladate(II) (Pb 37%) purchased from Alfa Aesar Co., Ltd., USA. Tri-sodium citrate dihydrate (99.5%) was supplied by Fisher Scientific Co., Ltd., USA. A commercial copper plating solution (Plug N Plate Cu solution) was offered by Caswell Inc., USA. Graphite (Gr, MA-EN-AN-03010F), lithium nickel cobalt manganese oxides (NCM811, MA-EN-CA-0B0307), lithium iron phosphate (LFP, MA-EN-CA-05), lithium cobalt oxide (LCO, MA-EN-CA-0Q), carbon black (Super-P, MA-EN-CO-01), polyvinylidene fluoride (PVDF, 5130, Solvay) were purchased from Canrd Co., Ltd., China.

*Preparation of MPETs*. At first, PET fabrics were treated with plasma for 30 mins. Then, fabrics were soaked in a silanization solution for 1 hour, which contains EtOH, acetic acid, deionized (D.I.) water, and MPTS with a volume ratio of 95:1:4:4. The carbon-carbon double bonds on the surface of treated fabrics provide sites for the polymerization of METAC, which can interact with the Pd^2+^ catalyst. The polymerization was conducted in a METAC solution (20% v/v in water) at 80 ℃ for 3 hours, by using potassium persulfate (2 g L^−1^) as initiator. At last, polymerized METAC-coated fabrics were dipped into a (NH_4_)_2_PdCl_4_ solution (5 mM ) for 30-60 min. The fabrics loaded with catalyst were then immersed into the ELD bath. Cu ELD process was realized by adding the reducing solution (HCHO, 36 mL L^−1^) into the Cu^2+^ source solution containing CuSO_4_·5H_2_O (13 g L^−1^), NaOH (12 g L^−1^), and KNaC_4_H_4_O_6_·4H_2_O (29 g L^−1^). Then, the electrodeposition was operated using CuPET as the working electrode and Cu foil as the counter electrode at a constant current density of 0.5 mA cm^-2^ with different plating time. ELD bath of Ni was conducted by adding the reducing solution of DMAB (0.75 g L^-1^) into the Ni^2+^ source solution containning Ni_2_SO_4_·5H_2_O (100 g L^-1^), sodium citrate (50 g L^-1^), lactic acid (25 g L^-1^) at the pH of ~7.5 (regulated by ammonium hydroxide). The ELD time is 60-90 min, depending on the target conductivity and areal density. ELD of Ni/P composite was carried out by changing the reducing agent and Ni^2+^ sources solution of the ELD bath. The source solution consists of Ni_2_SO_4_·5H_2_O (40 g L^-1^), sodium citrate (24 g L^-1^), and sodium acetate (24 g L^-1^) in DI water. The pH was regulated to ~4 by lactic acid. By adding the sodium hypophosphite solution (24g L^-1^) into the source solution, the ELD process was conducted at 85 ℃ for 60-90 min, depending on the target conductivity and areal density.

*Preparation of electrodes*. The active materials (Gr, NCM or LFP) were mixed with super P and PVDF with a weight ratio of 8:1:1 in a certain amount of NMP. Then, the slurry was blade-coated onto the metal foils or conductive fabrics. The samples were dried at 80 ℃, punched, compressed, and thoroughly dried under vacuum at 110 ℃.

*Assembly of cells.* Coin-type cells (CR2032) were assembled in an Ar-filled glovebox. Gr, NCM, LCO, and LFP electrodes were the working electrodes, and the counter electrodes were Li foils. Separator Celgard 2325 and electrolyte of 1M LiPF_6_ in EC/DEC/EMC solvent were used in all the cells. For the cell using the Gr electrode matched cathodes, the N/P ratio was controlled in the range of 1.1-1.2. Pouch cells were fabricated by sealing the electrodes into Al laminated composite film at Ar atmosphere. The cathode (~14 mg cm^-2^) and the anode (~9.2 mg cm^-2^) were cut into a rectangular shape (4 × 2 cm², the anode is a little bit larger than the cathode) and were welded with Al tab and Ni tab, respectively. The electrolyte addition is 60 μL.

*Characterizations.* Mechanical performances of current collectors were tested by a universal testing machine (Instron 5566, USA) at a cross speed of 50 mm min^-1^ and a gauge length of 20 mm. All the samples are cut into rectangular shapes of 40 mm×10 mm. The electrical properties of current collectors were characterized by a source meter (Keithley 2400, USA) with a four-point test probe (HPS58003, Helpass, China). Battery performances were obtained by a battery test system (BTS 3000, Neware, China). Electrochemical measurements were conducted on a minimum of three independent samples for each battery type, and representative data are presented. EIS, LSV, and CV tests were carried out by using an electrochemical workstation (Biologic, France). FTIR spectra were recorded by PerkinElmer FT-IR Spectrum 100 equipped with an attenuated total reflection (ATR) accessory. HPLC was tested by a 1260II system (Agilent, USA) equipped with a Fortis C18 column (4.6 × 250 mm). The mobile phase of methanol and 20 mM phosphate buffer was used at a flow rate of 0.5 mL min^-1^. The gradient elution was as follows: 0 to 15 min, 25% (v/v) methanol; 15 to 25 min, 25-100% (v/v) methanol liner gradient; 25 to 55 min, 100-25% (v/v) methanol liner gradient. The eluent was monitored at a wavelength of 240 nm. GC-MS was conducted in a united system (Agilent 7890/5975C, USA) and the protocol is the same as described by Petibon et al.^[1]^ The electrolyte was mixed with CH_2_Cl_2_ and filtered. After that, a small amount of water was added to extract salts. Then the aqueous layer was removed with a syringe and the residual water was binded with MgSO_4_. At last, the organic component was injected into the GC-MS.


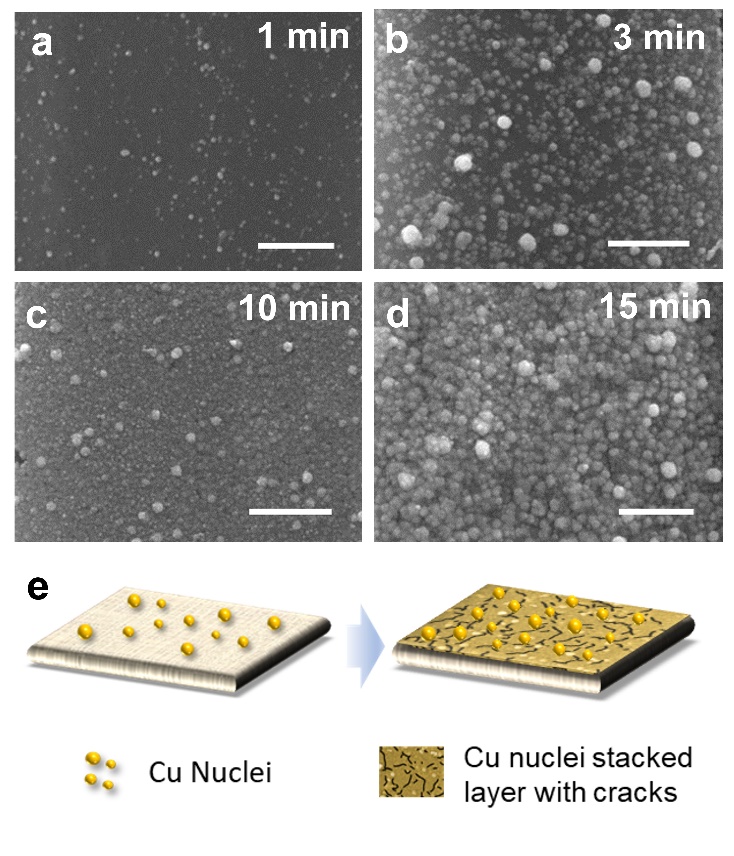


**Figure S1.** a-d) SEM images of Cu layer on the PET after ELD process with different time. e) Schematic process of electrodeless depostion.


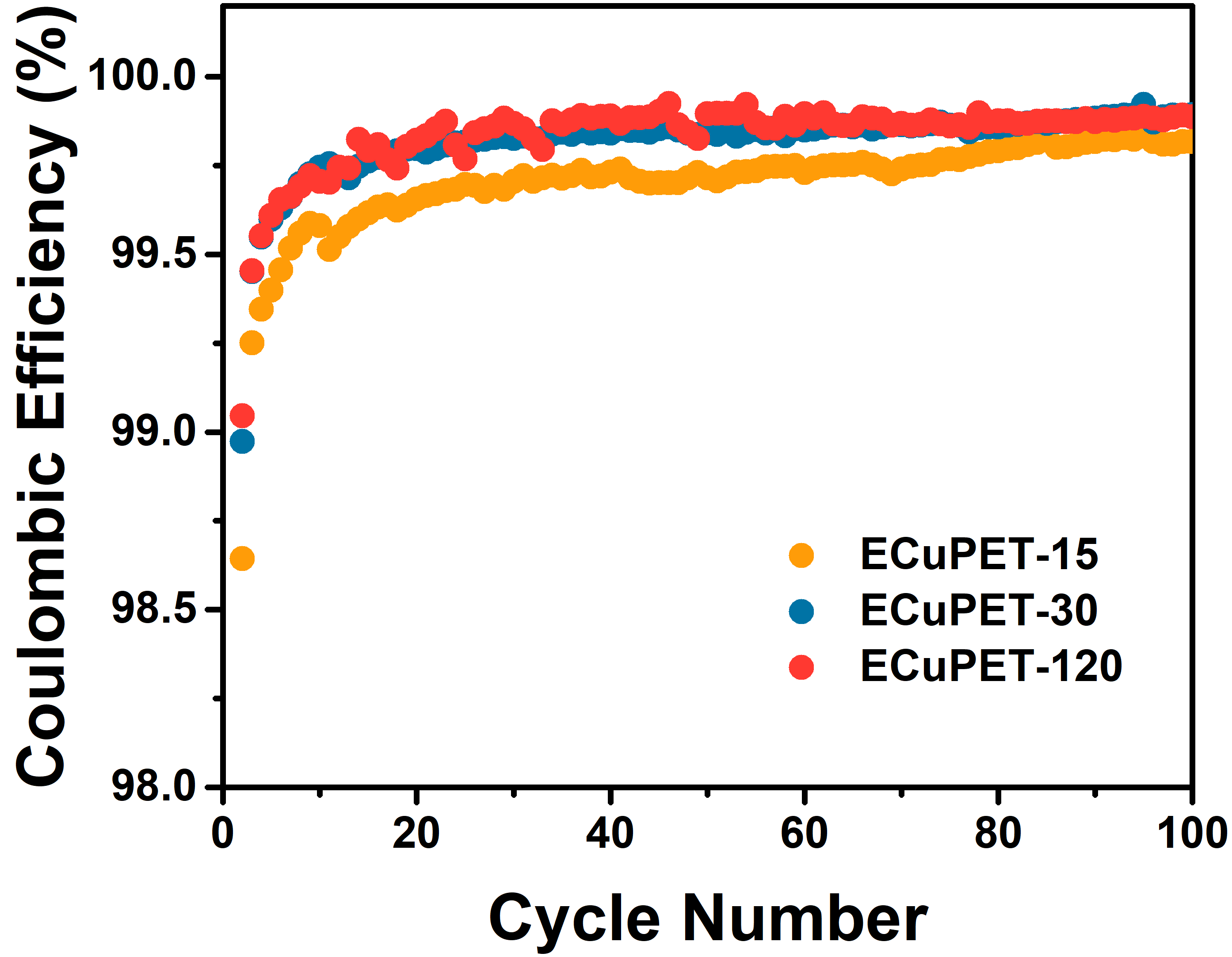


**Figure S2.** CEs of ECuPET with electroplating time of 15, 30 and 120 min at a current density of 0.5 mA cm^-2^.


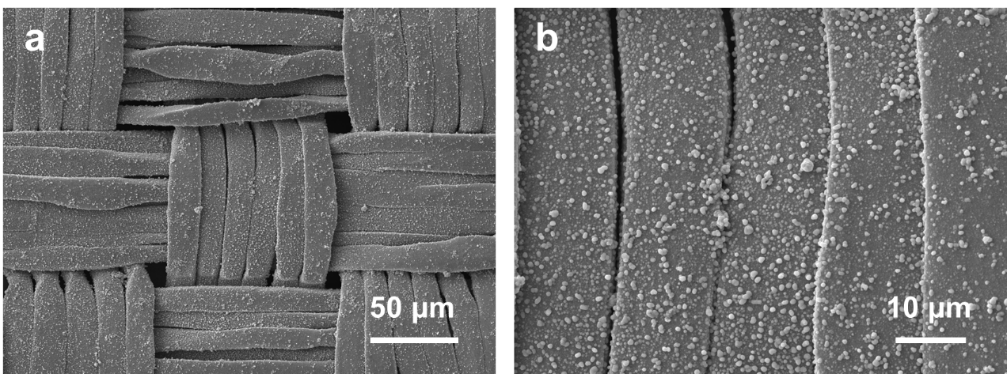


**Figure S3.** a) SEM image of ECuPET after 10000 bending cycles and b) partially enlarged image.


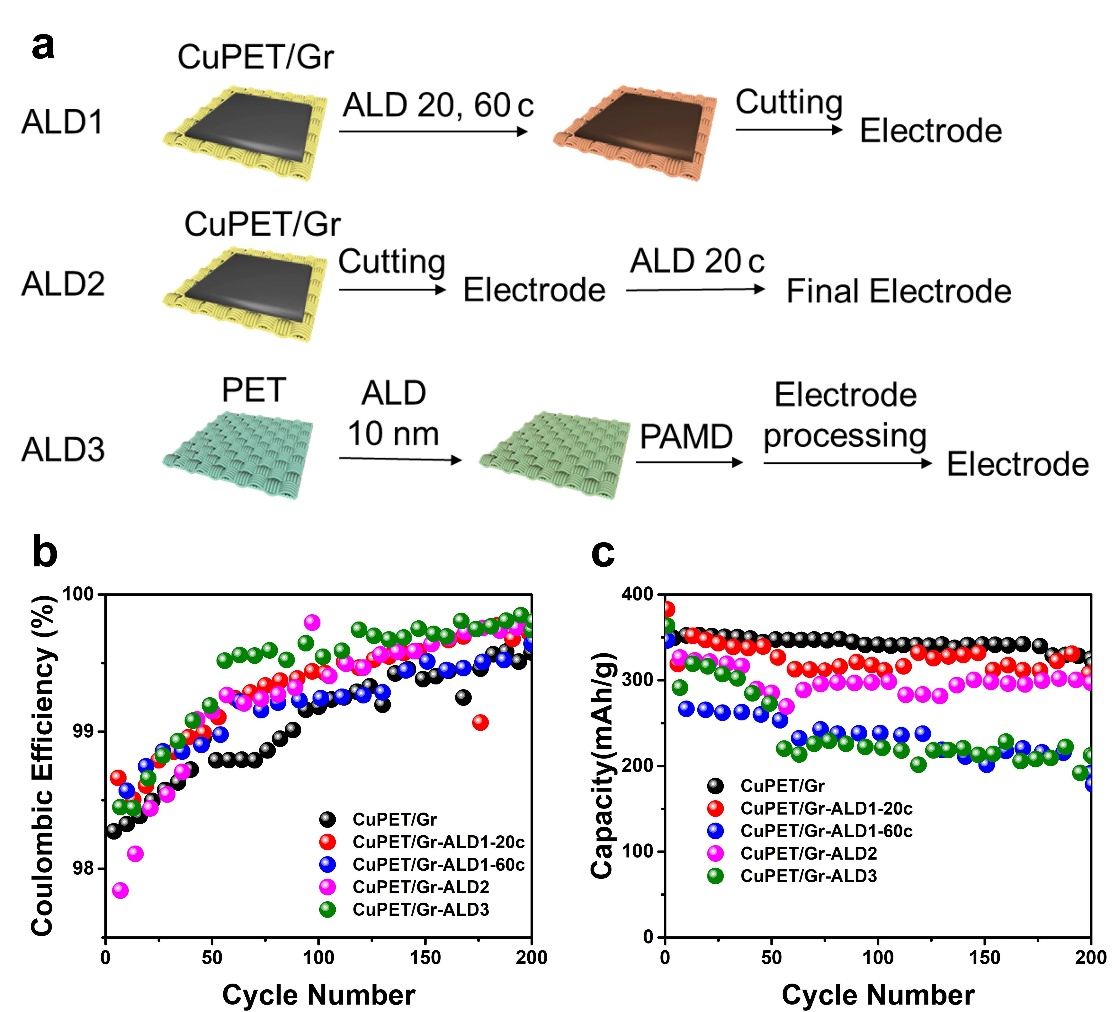


**Figure S4.** a) Schematic of different routes for ALD processes. b) Cycle performance of ALD-protected CuPET/Gr electrodes and c) corresponding CEs during cycling. The specific ALD process was conducted at 80 ℃ in a thermal ALD system (Savannah, Veeco), and the deposition thickness of every cycle in this condition is 0.75 Å. Samples with different routes and deposition cycles were named ALDx-yy (x=1, 2, 3 and yy indicates the deposition cycles). For example, ALD1-20 is the sample of CuPET treated with 20 ALD cycles in route 1.


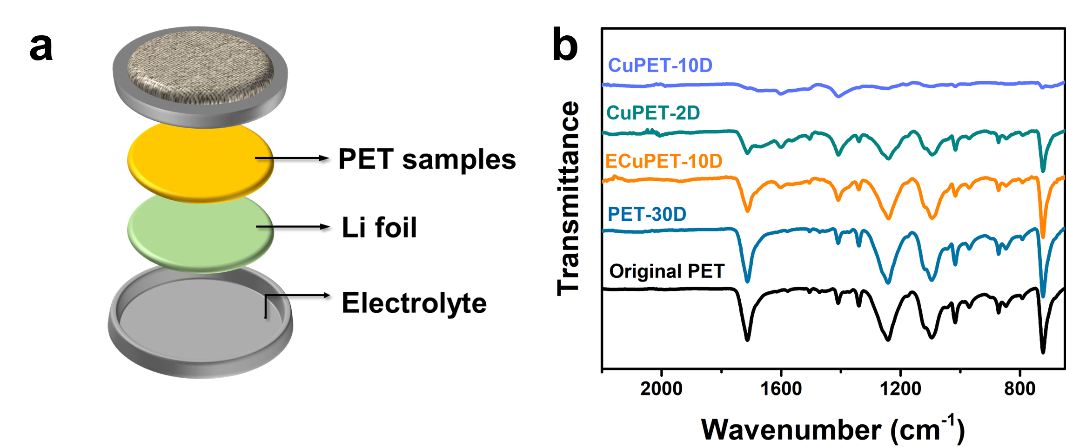


**Figure S5**. a) Schematic assembly for PET samples and Li foil in contact. b) FTIR results of PET samples in contact with Li foils for several days.


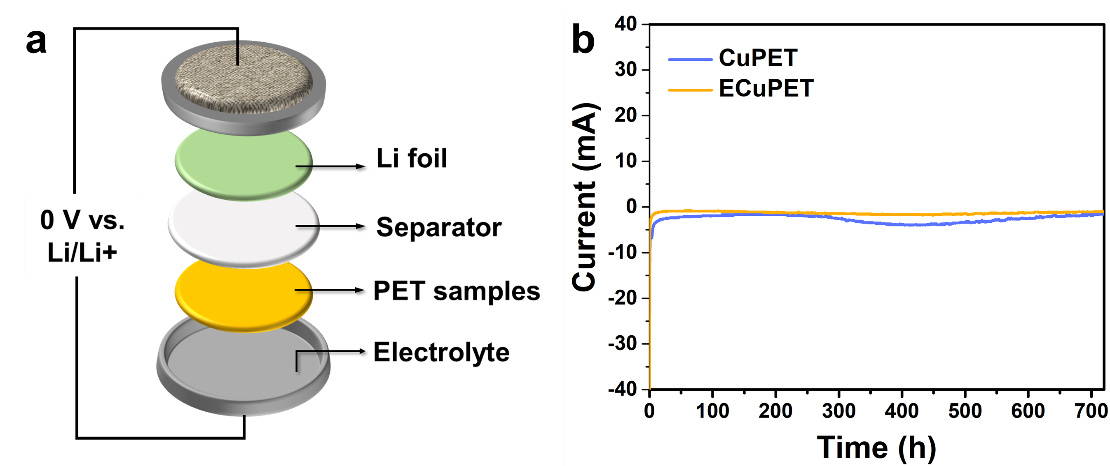


**Figure S6**. a) Schematic assembly for PET sample in a coin cell set at 0 V vs. Li/Li^+^. b) The leak current of CuPET and ECuPET at 0 V vs. Li/Li^+^.


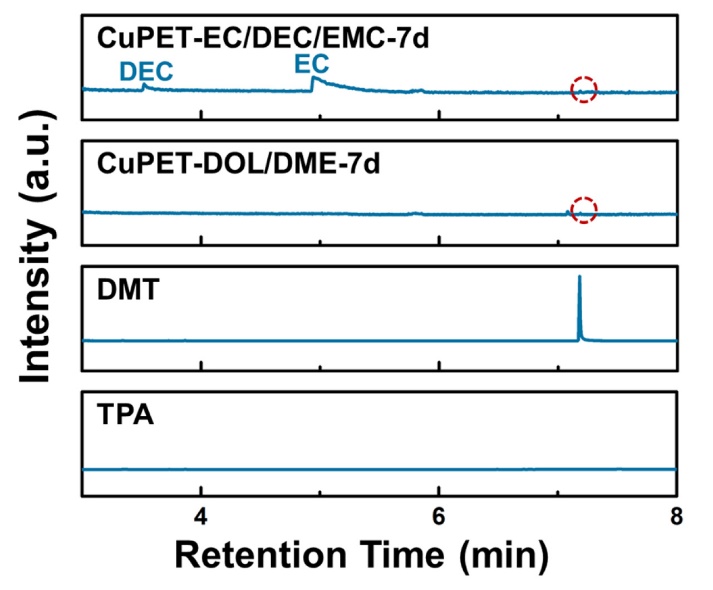


**Figure S7.** GC-MS curves of CuPET set at 0 V (vs. Li/Li+) in EC/DEC/EMC and DOL/DME electrolytes after seven days.


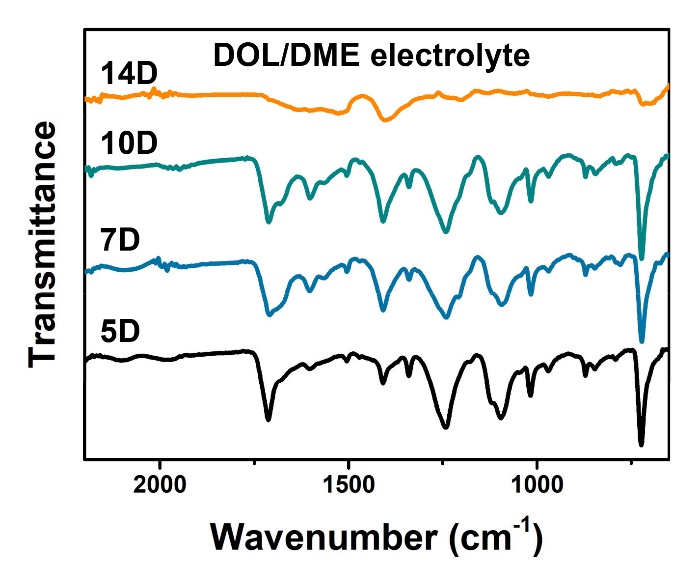


**Figure S8**. FTIR curves for CuPET set at 0 V (vs. Li/Li^+^) in the DOL/DME electrolyte for different days.


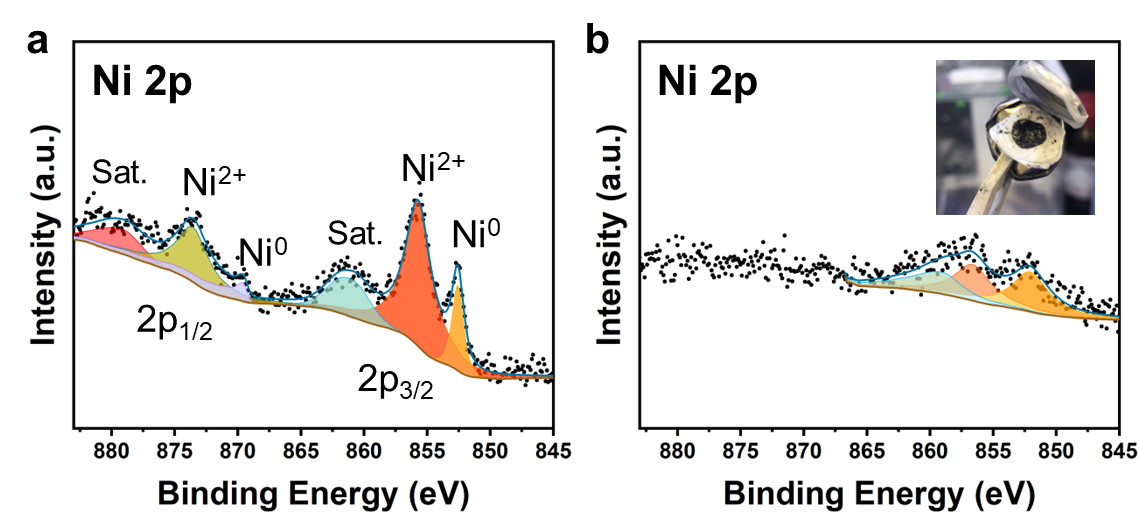


**Figure S9.** Ni 2p high-resolution XPS spectra of the NiPET a) before and b)after cycling. Inset of b) is the photograph of a disassembled coin cell using NiPET.


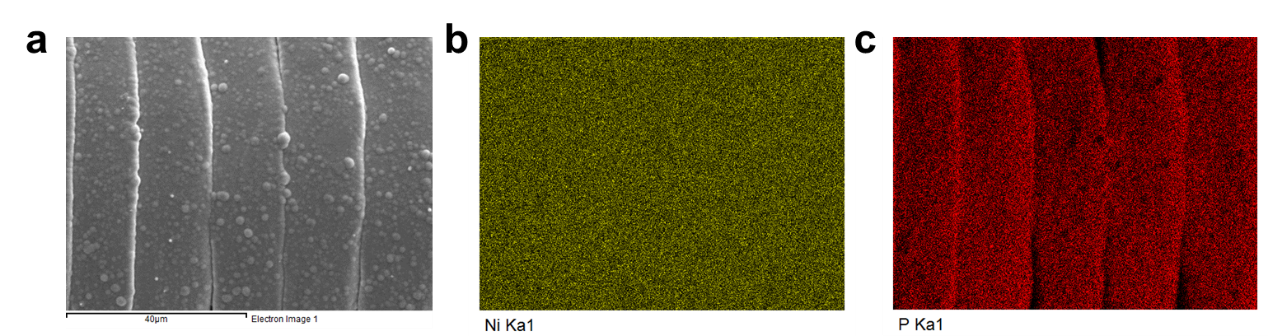


**Figure S10.** a）SEM image of NiPPET. Corresponding EDS mapping of b) Ni and c) P.


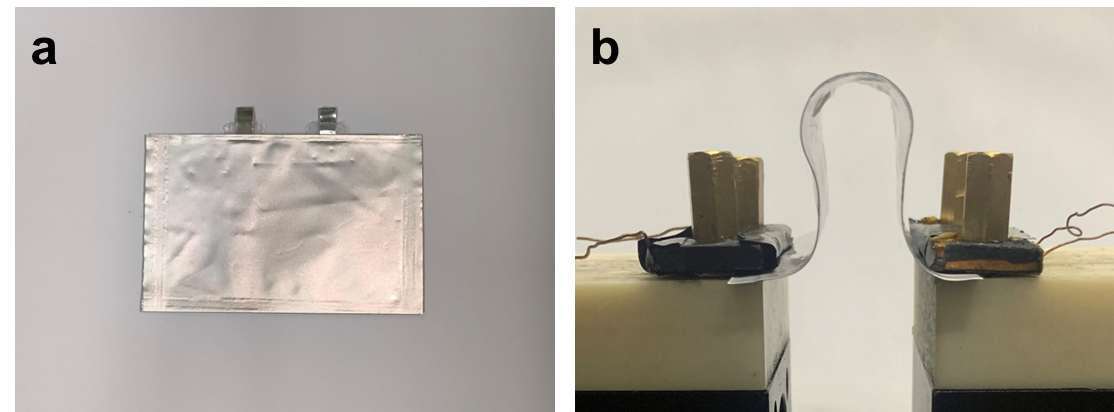


**Figure S11.** Photograph of the assembled pouch cell in a) the unbent state and b) the bent state.

**Table S1**. Comparison of different current collectors on areal density, sheet resistance, mechanical strength and electrochemical performance.

| **Current collector** | **Areal Density**  **(mg cm^-2^)** | **Sheet Resistance**  **(Ω cm^-2^)** | **Mechanical**  **Strength （MPa）** | **Assembly Type** | **Energy Density（Wh kg^-1^）** | **Ref.** |
| --- | --- | --- | --- | --- | --- | --- |
| ECuPET | 2.1 | 0.06 | 147.3 | ECuPET/Gr\|\|NiPPET/NCM | 219.9 | This work |
| NiPPET | 2.7 | 0.95 | 143.9 |  |  | This work |
| Cu foil | 7.5 | <0.01 | 308.0 | Cu/Gr\|\|Al/NCM | 182.7 | / |
| Al foil | 4.2 | <0.01 | 250.8 |  |  | / |
| CuGF | 2.9 | 0.11 | 146 | CuGF/Gr\|\|Al/NCM | 198 | ^[2]^ |
| NiGF | 3.2 | 0.45 | 168 |  |  | ^[2]^ |
| CC^a^ | / | / | / | preLi-CC^b^/Li\|\|Al/LFP | 167 | ^[3]^ |
| CC^a^ | 12.5 | 3.04 | ~2 | CuCC/Li\|\|NiCF/S | 288 | ^[4]^ |
| CuCC^a^ | 14.8 | 0.1 | ~5 |  |  | ^[4]^ |
| NiCC^a^ | 18.9 | 0.48 | ~8 |  |  | ^[4]^ |
| CNT^c^ film | 0.2 | 5 | / | CNT/LTO\|\|CNT/LCO | 108 | ^[5]^ |
| SWCNT^c^ film | 0.12 | 4 | / | / | / | ^[6]^ |
| CF^d^ film | 2.5 | 20 | / | / | / | ^[7]^ |
| Graphene film | 1.8 | ~10 | 32 | / | / | ^[8]^ |

a) “CC”, “CuCC” and “NiCC” represent carbon cloth, Cu-coated carbon cloth and Ni-coated carbon cloth. b) “preLi-CC” represents “prelithiated carbon cloth”. c). “CNT” and “SWCNT” represent carbon nanotube and single-walled carbon nanotube. d) “CF” represents carbon fiber.

**Table S2.** Calculation of energy density of batteries using metal foils and MPETs.

|  | Metal foils | MPETs |
| --- | --- | --- |
| Anode current collector (mg cm^-2^) | 7.5 | 2.1 |
| Graphite anode (mg cm^-2^) ^a^ | 9.4 | 9.1 |
| Cathode current collector (mg cm^-2^) | 4.2 | 2.7 |
| NCM cathode (mg cm^-2^) ^a^ | 13.9 | 14.4 |
| Electrolyte (mg cm^-2^) | 4.1 | 4 |
| Seperator (mg cm^-2^) | 1 | 1 |
| Total weight (mg cm^-2^) | 40.1 | 33.3 |
| Capacity (mAh cm^-2^) | 2.06 | 1.99 |
| *E_a_* (mWh cm^-2^) | 7.34 | 7.32 |
| Energy density (Wh kg^-1^) | 219.9 | 182.7 |

^a^ anode and cathode include binder and conductive agent.

**Table S3**. Calculated weight fractions of different battery components for Li-ion batteries.

|  | **Battery using metal foils** | | **Battery using MPETs** | |
| --- | --- | --- | --- | --- |
|  | Weight (mg) | Weight percent (%) | Weight (mg) | Weight percent (%) |
| Anode current collector | 3.75 | 12.6 | 1.05 | 4.0 |
| Graphite | 6.7 | 22.6 | 6.7 | 25.6 |
| Cathode current collector | 2.1 | 7.1 | 1.35 | 5.2 |
| NCM | 11.1 | 37.4 | 11.1 | 42.4 |
| Electrolyte | 4 | 13.5 | 4 | 15.3 |
| Seperator | 1 | 3.4 | 1 | 3.8 |
| PVDF | 0.5 | 1.7 | 0.5 | 1.9 |
| Carbon black | 0.5 | 1.7 | 0.5 | 1.9 |
| Total weight | 29.65 |  | 26.2 |  |

**Note:** The areal capacity is 2 mAh cm^-2^ and the weights of current collectors are halved in consideration of the double-side coating.

**Reference**

[1] R. Petibon, L. Rotermund, K. J. Nelson, A. S. Gozdz, J. Xia, J. R. Dahn, *Journal of The Electrochemical Society* **2014**, 161, A1167.

[2] J. Shang, W. Yu, L. Wang, C. Xie, H. Xu, W. Wang, Q. Huang, Z. Zheng, *Advanced Materials* **2023**, 35, 2211748.

[3] C. Xie, J. Chang, J. Shang, L. Wang, Y. Gao, Q. Huang, Z. Zheng, *Advanced Functional Materials* **2022**, 32, 2203242.

[4] J. Chang, J. Shang, Y. Sun, L. K. Ono, D. Wang, Z. Ma, Q. Huang, D. Chen, G. Liu, Y. Cui, Y. Qi, Z. Zheng, *Nature Communications* **2018**, 9, 4480.

[5] L. Hu, H. Wu, F. La Mantia, Y. Yang, Y. Cui, *ACS Nano* **2010**, 4, 5843.

[6] R. Fang, G. Li, S. Zhao, L. Yin, K. Du, P. Hou, S. Wang, H.-M. Cheng, C. Liu, F. Li, *Nano Energy* **2017**, 42, 205.

[7] Y. Wang, K. Chen, *Journal of Electroanalytical Chemistry* **2019**, 849, 113374.

[8] Y. Shi, L. Wen, G. Zhou, J. Chen, S. Pei, K. Huang, H.-M. Cheng, F. Li, *2D Materials* **2015**, 2, 024004.
